# MicroRNA-1 Accelerates the Shortening of Atrial Effective Refractory Period by Regulating *KCNE1* and *KCNB2* Expression: An Atrial Tachypacing Rabbit Model 

**DOI:** 10.1371/journal.pone.0085639

**Published:** 2013-12-30

**Authors:** Xiaomeng Jia, Shaohua Zheng, Xinxing Xie, Yujiao Zhang, Weizong Wang, Zhongsu Wang, Yong Zhang, Jiangrong Wang, Mei Gao, Yinglong Hou

**Affiliations:** Department of Cardiology, Shandong Provincial Qianfoshan Hospital, Shandong University, Jinan, Shandong, China; Loyola University Chicago, United States of America

## Abstract

**Background:**

The potential mechanisms of *microRNA-1* (miR-1) in the electrical remodeling of atrial fibrillation remain unclear. The purpose of this study was to evaluate the effects of *miR-1* on the atrial effective refractory period (AERP) in a right atrial tachypacing model and to elucidate the potential mechanisms.

**Methods and Results:**

QRT-PCR and western blot were used to detect the expression of the *miR-1*, *KCNE1*, and *KCNB2* genes after 1-week of right atrial tachypacing in New Zealand white rabbits. The AERP was measured using a programmable multichannel stimulator, and atrial fibrillation was induced by burst stimulation *in* *vivo*. The slowly activating delayed rectifier potassium current (*IKs*) and AERP in atrial cells were measured by whole cell patch clamp *in* *vitro*. Right atrial tachypacing upregulated *miR-1* expression and downregulated *KCNE1* and *KCNB2* in this study, while the AERP was decreased and the atrial *IKs* increased. The downregulation of *KCNE1* and *KCNB2* levels was greater when *miR-1* was further upregulated through *in* *vivo* lentiviral infection. Electrophysiological tests indicated a shorter AERP, a great increase in the *IKs* and a higher atrial fibrillation inducibility. In addition, similar results were found when the levels of *KCNE1* and *KCNB2* were downregulated by *small* *interfering* *RNA* while keeping *miR-1* level unaltered. Conversely, knockdown of *miR-1* by anti-miR-1 inhibitor oligonucleotides alleviated the downregulation of *KCNE1* and *KCNB2*, the shortening of AERP, and the increase in the *IKs*. *KCNE1* and *KCNB2* as the target genes for *miR-1* were confirmed by luciferase activity assay.

**Conclusions:**

These results indicate that *miR-1* accelerates right atrial tachypacing-induced AERP shortening by targeting potassium channel genes, which further suggests that *miR-1* plays an important role in the electrical remodeling of atrial fibrillation and exhibits significant clinical relevance as a potential therapeutic target for atrial fibrillation.

## Introduction

Atrial fibrillation (AF) is one of the most common arrhythmias and is associated with a substantial morbidity, mortality, and socioeconomic burden [1]. Experimental and clinical studies have demonstrated that electrical remodeling (ER) and structural remodeling play major roles in AF. ER occurs early during AF and leads to characteristic changes in the atrial effective refractory period (AERP) and a loss of rate adaptation [2]. Recently, the abnormal expression of genes encoding ion-channel proteins, especially the potassium (K^+^) channel, has attracted interest in the molecular mechanism underlying these AERP changes and the vulnerability to AF. Differences between messenger ribonucleic acid (mRNA) levels and the corresponding proteins have been observed frequently in gene expression studies [3], and this regulatory mechanism at the post-transcriptional level suggests that microRNAs (miRNAs) should play an important role in AF. 

MiRNAs comprise a group of endogenous single-stranded non-protein-coding small RNAs (~ 22 nucleotides long) that were initially described in 1993 [4]. MiRNAs interact with the 3' untranslated region (3'UTR) of their target mRNAs via perfect or imperfect complementarity with 2–8 nt at their 5' end, which is the ‘seed sequence’ that is critical for miRNA actions [5] to guide RNA- induced silencing complex (RISC) to down-regulate the expression of the target mRNA at the post-transcriptional level. 

*MiRNA-1* (*miR-1*) is a muscle-specific miRNAs that is preferentially expressed in adult cardiac and skeletal muscle tissues [6], and is among the top 20 most abundant miRNAs in human heart tissue [7,8]. Current studies indicate that *miR-1* is involved in many heart diseases, especially in cardiac arrhythmias, and its expression is associated with cardiac arrhythmogenic potential in ischemic heart diseases [9]. Delivery of *miR-1* into normal or infarcted rat hearts induces a significantly widened QRS complex and prolonged QT interval in electrocardiograms and *AMO-1* (anti-miR-1 inhibitor oligonucleotides) reverses this effect. The upregulation of *miR-1* increases conduction time and depolarizes membrane potential via repression of *Kir2.1* and *Connexin 43* level, which might be partially responsible for the arrhythmogenic potential of *miR-1*. Terentyev et al. [10] suggested recently that *miR-1* also participates in arrhythmia via the impairment of *Ca2* ^+^ handling.

The rabbit *miRNA* profile has been absent from miRbase until now. The role of *miR-1* in the pathogenesis of cardiac arrhythmia has been studied, but its potential role in AERP changes following atrial tachypacing (A-TP) in rabbit has not been investigated. Therefore, the present study investigated the *miR-1* accelerated the AERP shortening following 1-week of A-TP in a rabbit model. In this work, we used lentiviral vectors (LVs) to deliver the genes of interest. LVs exhibit low immunogenicity and they are easily infected into cells and tissues, which leads to higher gene expression levels than the adenovirus vectors used in similar previous studies [11-15]. 

## Materials and Methods

### Ethics statement

The use of animals and all procedures were performed in accordance with the regulations of the *Guide for the Care and Use of Laboratory Animals* published by the United States National Institutes of Health (NIH publication no. 85-23, revised 1996), and approved by the Animal Care and Use Committee of Shandong University. 

### A-TP model

Adult New Zealand white rabbits (both genders; 1.5-2.5 kg) were randomly allocated into 6 groups: a control group (Ctl, n=6), no pacing but infected with control LVs; a right A-TP group (Pacing, n=6), submitted to pacing at 600 beats per minute (bpm) for 1 week and subsequently infected with control LVs; a right A-TP infected with *miR-1* group (P + miR-1, n=6), recombinant LVs carrying *miR-1* injected into the right atrium (RA) after right A-TP; a right A-TP infected with *AMO-1* group (P + *AMO*-1, n=6), recombinant LVs carrying *AMO-1* injected into the RA after right A-TP; a right A-TP infected with small interfering RNA (siRNA)*-KCNE1* group (P + *siR-KCNE1*, n=6), recombinant LVs carrying *siR-KCNE1* injected into the RA after right A-TP; and a right A-TP infected with *siR-KCNB2* group (P + *siR-KCNB2*, n=6), recombinant LVs carrying *siR-KCNB2* injected into the RA after right A-TP. Rabbits were anesthetized with sodium pentobarbital (30–35 mg/kg) and ventilated by tracheostomy using a volume-regulated respirator (Shimano, model SN-480-5, Tokyo, Japan). Halothane (2–3%) and N_2_O (60–75%) were supplemented to maintain a constant level of anesthesia during all procedures [11]. The ventilator settings (tidal volume 15-40 ml, respiratory rate 30-50 bpm, and inspiratory-to-expiratory ratio of 2:1) were adjusted to maintain physiological arterial blood gases (pH 7.35–7.45, PO_2_ 80–120 Torr, and PCO_2_ 25–40 Torr) every 2 h [12]. After the administration of local anesthesia using lidocaine (1%, 3-5ml) [11] into the neck skin, the right jugular vein was isolated and ligated by skin incision. A pacemaker (AOO, made in Shanghai Fudan University, China) was implanted in a subcutaneous pocket and attached to an electrode-lead in the RA appendage via the right jugular vein under X-ray guidance [13]. All surgical procedures were performed under sterile conditions.

### Electrophysiological monitoring *in vivo*

An electrophysiological examination was performed at 3 time points (before pacing, before infection and 1-week after infection), using a programmable multichannel stimulator (model DF-5A Electrophysiology; Jinjiang Co., Sichuan, China), and intracardiac electrograms (ECG) were measured using an electrophysiological recording system by placing a catheter into the RA. The AERP was measured using S1-S2 programmed electrical stimulation (PES) [14], S2 at coupling intervals starting at 130 ms, and progressively shortened at 5 ms decrements with a drive cycle length of 150 ms. The AERP was defined as the longest Sl-S2 interval that failed to produce a response.

AF was induced by PES with burst stimulation (cycle length 80 ms, lasting 2-3 minutes). The pacing amplitude was set at twice the diastolic threshold. Successful induction of AF was defined as a period of rapid irregular atrial rhythms lasting at least 30 seconds. 

### Atrial myocyte cellular isolation and electrophysiological recording

Atrial myocyte isolation: after the rabbits were euthanized, the heart was immediately excised and washed by ice-cold PBS retroperfusion via the aortic root, tied with langendorff perfusion system by suture, and perfused with Ca^2+^-free Tyrode’s solution (36°C; containing [mmol/l]: NaCl 118, KCl 4.8, MgCl_2_ 1.25, CaCl_2_ 1.5, NaH_2_PO_4_ 1.25, MgSO4 1.25, 4-(2-hydroxyethyl)-1-piperazineethanesulfonic acid (HEPES) 10, and D-glucose 11; pH 7.4 with NaOH). Perfusate was then switched to Ca^2+^-free Tyrode’s solution (NaCl 118, KCl 4.8, MgCl_2_ 1.25, NaH_2_PO_4_ 1.25, MgSO4,1.25, 4-(2-hydroxyethyl)-1-piperazineethanesulfonic acid (HEPES) 10, and D-glucose 11; pH 7.4 with NaOH) containing collagenase TypeⅡ(200 U/ml) and protease type XIV (0.1%) for 20–30 min followed by a 4 min washout with Ca^2+^ free Tyrode’s solution. The heart will be swollen and extremely digested. All perfusates were gassed with 100% O_2_ and maintained at 37°C. The RA myocardium was gently cut into small pieces and then incubated for 10 min. After gauze filtration, dispersed myocytes were resuspended in sequentially higher Ca^2+^ concentrations, and stored at room temperature for study within 2 h of isolation. 

Cellular electrophysiological recording: Conventional whole cell patch clamp was performed as described previously [16,17]. The membrane currents were recorded with whole cell patch clamp in voltage clamp mode, and membrane potentials were recorded in current clamp mode. The borosilicate-glass electrodes had tip resistances between 2 and 5 MΩ. Cell capacitance and series resistance were compensated by approximately 80% to 90%. Currents are expressed as current density (normalized to the cell capacitance). For AERP recording, nystatin (60 g/mL) was back-filled into the pipette tip [K-aspartate 110, KCl 20, MgCl_2_ 1, MgATP 5, GTP 0.1, HEPES 10,Na-phosphocreatine 5, and EGTA 5 (for current recording) or 0.025 (for AERP recording) ( pH 7.3 with KOH)], and the external solutions contained 2 mmol/L CaCl_2_. For all *K* ^+^ current recordings, CdCl_2_ (0.1 mol/L) was added to the external solutions to block Ca^2+^ currents. E4031(1 μmol/L) was included in the extracellular solution for the slowly activating delayed rectifier potassium current (*IKs*) measurement. The *IKs* was recorded by a holding potential of -80mV following a depolarization from -60mV to 60mV stepped by a 10mV for 5s, then a repolarization potential to -30mV for 2s. All experiments were performed at room temperature. The statistical analysis was performed using Origin 7.0. 

The AERP was determined using a train of 8 conditioning current pulses (S1), S2 at coupling intervals starting at 200 ms. The S1–S2 interval was shortened by 10 ms intervals and the ERP was defined as the longest S1–S2 interval which failed to elicit action potentials [18]. 

### Construction of the LVs

The *miR-1* sequence of the New Zealand white rabbit was obtained from the Sanger Institute ENSEMBL Sequence Database according to the draft genome sequence (http://asia.ensembl.org/Oryctolagus_cuniculus/Info/Index) (Table 1). The *miR-1* precursor DNA (5'-ACCTACTCAGAGTACATACTTCTTTATGTACCCATATGAACATACAATGCTATGGAATGTAAAGAAGTATGTATTTTTGGTAGG-3'), the *siR-KCNE1* (5’-GGAGAAAGACAGGGCGTACTT-3’), and *siR-KCNB2* (5’-GCAGGAAACGGATGAATTTGG-3’) were synthesized by TELEBIO (Shanghai, PR China). The shRNA fragment was inserted into a third generation LV. Recombinant LV-*miRNA*-*1*, LV-*siR-KCNE1* and LV-*siR-KCNB2* plasmids were isolated and purified after verified by double-restriction enzyme digestion and gene sequence analysis. Five nucleotides at the end of the *AMO-1* gene, which were the exact antisense copies of the mature *miRNA* sequence, were locked (the ribose ring was constrained by a methylene bridge between the 2'-O- and the 4'-C atoms).

**Table 1:** MiR-1 sequences.

Name	Species	Accession no.	Sequence
ocu-mir-1	rabbit	NSCUT00000020310	5'-UGGAAUGUAAAGAAGUAUGUAU-3'
mmu-mir-1a	mouse	MI0000139	5'-UGGAAUGUAAAGAAGUAUGUAU-3'
has-mir-1	human	MI0000651	5'-UGGAAUGUAAAGAAGUAUGUAU-3'
ron-mir-1	rat	MI0003489	5'-UGGAAUGUAAAGAAGU **G**UGUAU-3'
cfa-mir-1-1	dog	MI0008060	5'-UGGAAUGUAAAGAAGUAUGUA-3'

The mature sequences of miRNA are highly conserved, which suggests that miRNAs have the same basic mechanism in the development process different species’. The rabbit, mouse, and human have the same mature sequence, and there is only one different nucleotide (the bolded squared) in the sequence of the rat and the dog.

### LVs production

Four plasmids (pRSV-Rev, pMDLg-pRRE, pMD2G, and interference plasmid) were co-transfected-into HEK293T cells. The DNA-containing medium was replaced with fresh medium containing 20% fetal bovine serum 8 hours later. LV particles were collected from the cell supernatant after 48 hours of incubation and purified and concentrated using ultra-centrifugation. The final preparations were obtained with a titer of 1 × 10^9^ TU/ml for injection into the rabbits.

### *In vivo* gene infection

A thoracotomy was performed in the right fourth intercostal space after 1-week of A-TP, and the heart was exposed after excising the pericardium. The RAs were fixed, and 80 μl of recombinant LVs were directly injected into 8 separate sites to deliver the vector particles uniformly over a large injection area [9]. Subsequently, the heart was replaced into the thoracic cavity, the chest was closed with sutures, and a syringe was used to evacuate the air/fluid in the thoracic cavity to regain the normal negative physiological intrathoracic pressure. The rabbits were euthanized using an intraperitoneal injection of sodium pentobarbital (150 mg/kg) 1- week after the lentiviral injection. The heart was immediately excised and perfused. The RA was dissected, and part of it was used for electrophysiological recordings, and the other part was blotted dry, frozen in liquid nitrogen, and stored at -80° C until use for the detection of *miR-1* gene expression and mRNA and protein quantification (see below).

### Quantitation of mRNA and *miR-1* levels

Total RNA was extracted from rabbit RAs by homogenizing 50-100 mg of tissue in 1 ml TRIzol® Reagent (Invitrogen) using a TissueRuptor (QIAGEN) according to the manufacturers' instructions. cDNA was synthesized by reverse transcribing the total RNA obtained from each sample. The complete reaction mixture (RM, 25μl) contained 2 μg RNA, 1μl random primer/mir-1 specific reverse transcript primer (Table 2), 10μl DEPC-H_2_O, 5 μl 5 × reverse transcriptase (RT) buffer, 5 μl dNTPs, 1 μl RTs, and 1 μl RNase inhibitor. Three of the above components, i.e., RNA, random primer, and DEPC-H_2_O (termed “RM1”), were incubated at 70° C for 5 min and chilled on ice for 5 min. The 4 our other components of the RM, i.e., the RT buffer, dNTPs, RT, and RNase inhibitor (termed “RM2”), were incubated at 65° C for 5 min. Subsequently, RM1 and RM2 were mixed and incubated for 60 min at 37° C and heated at 90° C for 5 min. Thereafter, quantitative real-time reverse-transcription polymerase chain reactions (qRT-PCRs) were performed using an ABI 7900a instrument. Reactions (20 μl) contained 1 μl cDNA and 5 μM of each forward and reverse primer (Table 2). The following PCR parameters were used: 3 min at 95° C, followed by 45 cycles of 15 s at 95° C, 20 s at 60° C, and 15 s at 72° C. The PCR tube for the *miR-1* reactions (20 μl) contained 1.1 μl cDNA and 5 μM of each forward and reverse primer (Table 2). A 3-min denaturation step was followed by 40 cycles of 15 s at 94° C, 20 s at 60° C, and 15 s at 72° C. The relative levels of each product were normalized according to the ^∆∆^C (t) method.

**Table 2:** The primers for qRT-PCR.

Gene	Accession no.	Sequence
*miR-1*	NSCUT00000020310	F:5'--CTCAACTGGTGTCGTGGAGTCGGCAATTCAGTTGAGATACACAC--3'
		R:5'--ACACTCCAGCTGGGTGGAATGTAAAGAAGT--3'
*β-actin*	NM_001101683.1	F:5'- -ACCACAGTCCATGCCATCAC--3'
		R: 5'--TCCACCACCCTGTTGCTGTA--3'
*KCNB2*	NM_001082137.1	F: 5'--CCTGGCGCTGCGGCTTTGTCC--3'
		R: 5'-- AGGCGTGTCCGGGGCAGTCG--3'
*KCNE1*	NM_001109822.1	F: 5'--AACCGCCCGGACCCCAACAC--3'
		R: 5'--TTCCCGGACACGATGCCACAGG--3'

F, forward primer; R, reverse primer

### Western blots

The protein samples were obtained from the same rabbits RA samples that were used for RNA isolation, but the proteins were isolated with an extraction buffer containing 1ml RIPA containing 0.1mM PMSF, and then homogenized. After centrifugation at 1,000 rpm at 4°C for 5 min, and the supernatants containing cell membranes were further centrifuged at 12,000 rpm for 1 hour. Pellets were resuspended in extraction buffer containing 1% Triton and stored at -80° C. The protein content was determined using the BCA Protein Assay Kit (BCA, Haimen, ON, China) using bovine serum albumin as a standard. Membrane protein samples (~30 mg) were denatured using Laemmli buffer, resolved using SDS–PAGE (7% polyacrylamide gels), and transferred electrophoretically to Immobilon-P polyvinylidene fluoride membranes (Millipore, Bedford, MA) at 10 V for 1 hour in 0.3% Tris-base, 0.06% glycine, and 20% ethanol, pH 8.3. The membranes were blocked in Tris-buffered saline with 0.2% Tween 20 and 5% skim milk powder, and incubated with primary antibodies [mouse monoclonal anti-KCNE1 antibody (1:500, from Abcam), or mouse monoclonal anti-KCNB2 antibody (1:500, from Santa Cruz)] at 4°C overnight. The following day, the membranes were washed in TBST 5 times (for 5 min each time) and incubated for 2 h with horseradish peroxidase-conjugated monoclonal mouse anti-glyceraldehyde-3-phosphate dehydrogenates (GAPDH; 1:10,000, KangChen) in blocking buffer. The antibody was detected using Immobilon Western Chemiluminescent HRP Substrate (Millipore, Billerica, MA). Membranes were also probed with anti-GAPDH antibody from KangChen (Shanghai, China) as an internal standard to correct for difference in protein loading. Western blot bands were quantified using QuantityOne software by measuring the band intensity (Area × OD) for each group and normalizing to the observed GAPDH levels. The final results are expressed as fold changes by normalizing the data to the control values. 

### Luciferase reporter assays

The DNA fragment of the *KCNE1*/*KCNB2* 3'UTR was amplified from a rabbit cDNA library and cloned downstream of the luciferase gene to obtain the luc-*KCNE1*/*KCNB2* construct. The target sites were duplicated by PCR subcloning a portion of the *KCNE1*/*KCNB2* 3'UTR directly into the initial *KCNE1*/*KCNB2* construct to create luc-*KCNE1*/*KCNB2*. We synthesized fragments containing the exact target sites for *miR-1* and inserted them downstream of the luciferase gene. We generated miRNA sensor constructs of perfectly complementary sequences to *miR-1* directly downstream of the luciferase gene to confirm *miR-1* expression in the reporter assays. Reporter assays were conducted using HEK293T cells in triplicate in 24-well plates and replicated 3 times. Infections were performed with 50 ng of the reporter plasmid and 50, 100, or 150 ng of *miRNA* plasmids. A CMV-lacZ reporter was used as an internal control to normalize for infection efficiency, and the total amount of DNA per well was maintained at a constant value via the addition of the appropriate amount of empty expression vector. Nucleotide-substitution mutations were performed using direct oligomer synthesis for *miR-1* (MT *miR-1*) and PCR-based methods for the 3'-UTRs of *KCNE1* and *KCNB2*. MT *miR-1*: 5’-AUACAUACUUCUUUUGUAAGGA-3’; MT 3'UTR of *KCNE1*: 3’-UAUCAGUUCUGGACAUUCCCAUUGUUUGU-5’; MT 3'UTR of *KCNB2*: 3’-UUGAGUGUUGUGGACAUUCCAUUUUGGGGGUA-5’. All constructs were sequence verified. The underlined nucleotides indicate the bases where mutations were made.

### Data analysis

The statistical analysis was performed using SPSS 10.5 software. Data are presented as the means ± standard deviation (SD). The differences among multiple groups were assessed using one-way analysis of variance (ANOVA), and a Tukey’s post-hoc test was used to evaluate the significance of the differences between two groups. A two-tailed *P* < 0.05 was taken to indicate a statistically significant difference. 

## Results

### Hemodynamic parameters

Hemodynamic parameters (e.g., heart rate, systolic blood pressure, diastolic blood pressure, left ventricular systolic pressure, and left ventricular end-diastolic pressure) were assessed by heart catheterization 1-week after lentiviral injections to demonstrate the stability of our experiment, and no significant differences between the 6 groups were observed (P < 0.05) (Table S1).

### The effects of A-TP

**Figure 1:**
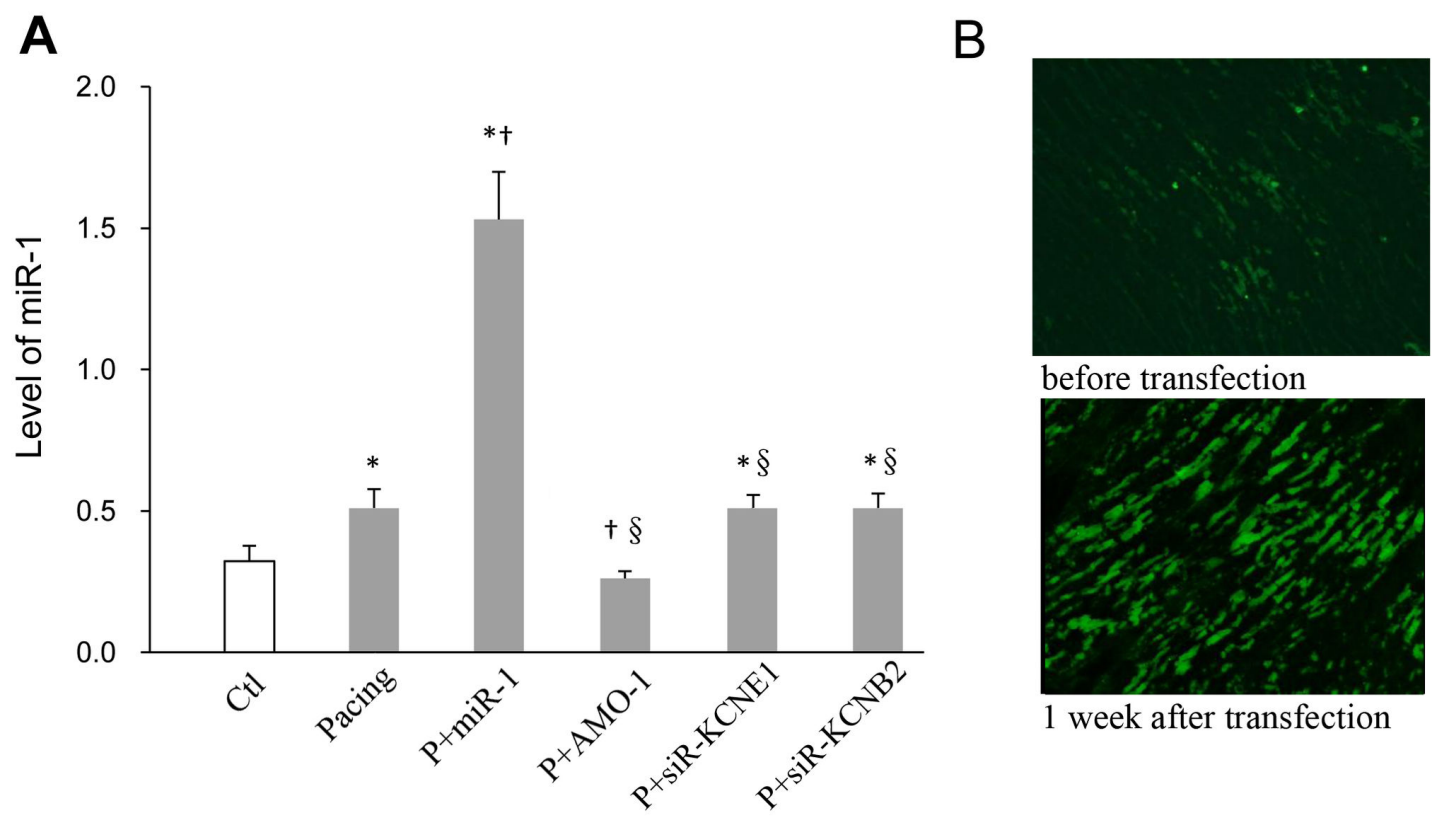
miR-1 expression. QRT-PCR quantification of *miR-1* in each group (A). Immunofluorescence analysis of atrial samples from the rabbit model of right A-TP obtained 1-week after LVs infection with *miR-1*-LVs (B). * *P* < 0.05 vs. Ctl, † *P* < 0.05 vs. Pacing, §*P* < 0.05 vs. P+miR-1, one-way ANOVA and Tukey’s post-hoc test; n = 6 independent RNA samples for each group.

**Figure 2:**
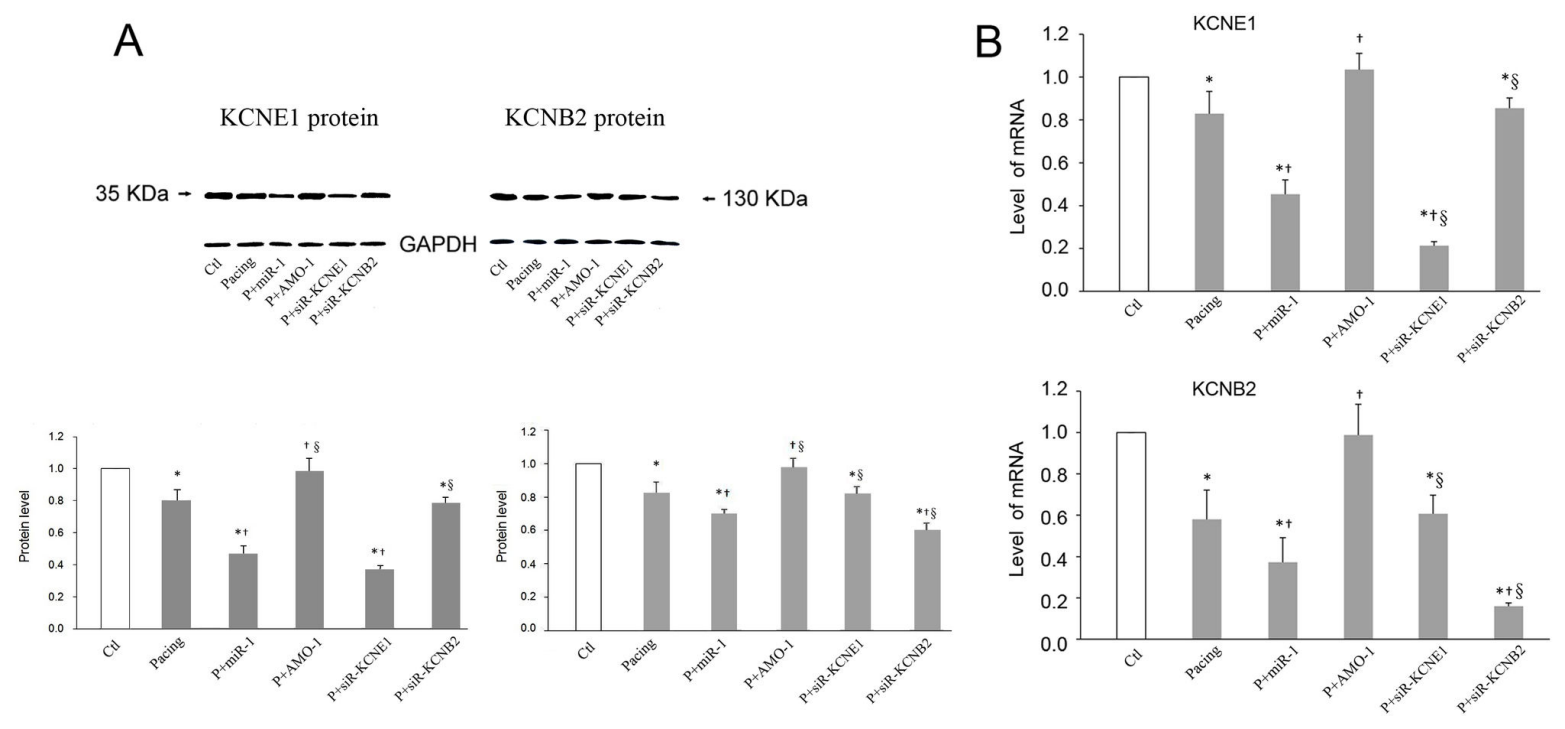
Evaluation of KCNE1 and KCNB2 expression. Western blot profiles (A) and qRT-PCR (B) of KCNE1 and *KCNB2* proteins levels (33 kDa and 132 kDa, respectively) and mRNAs levels, isolated from rabbits RA. * *P* < 0.05 vs. Ctl, † *P* < 0.05 vs Pacing, §*P* < 0.05 vs. P+miR-1. One-way ANOVA and Tukey’s post-hoc test; n = 6 independent RNA samples for each group.

**Figure 3:**
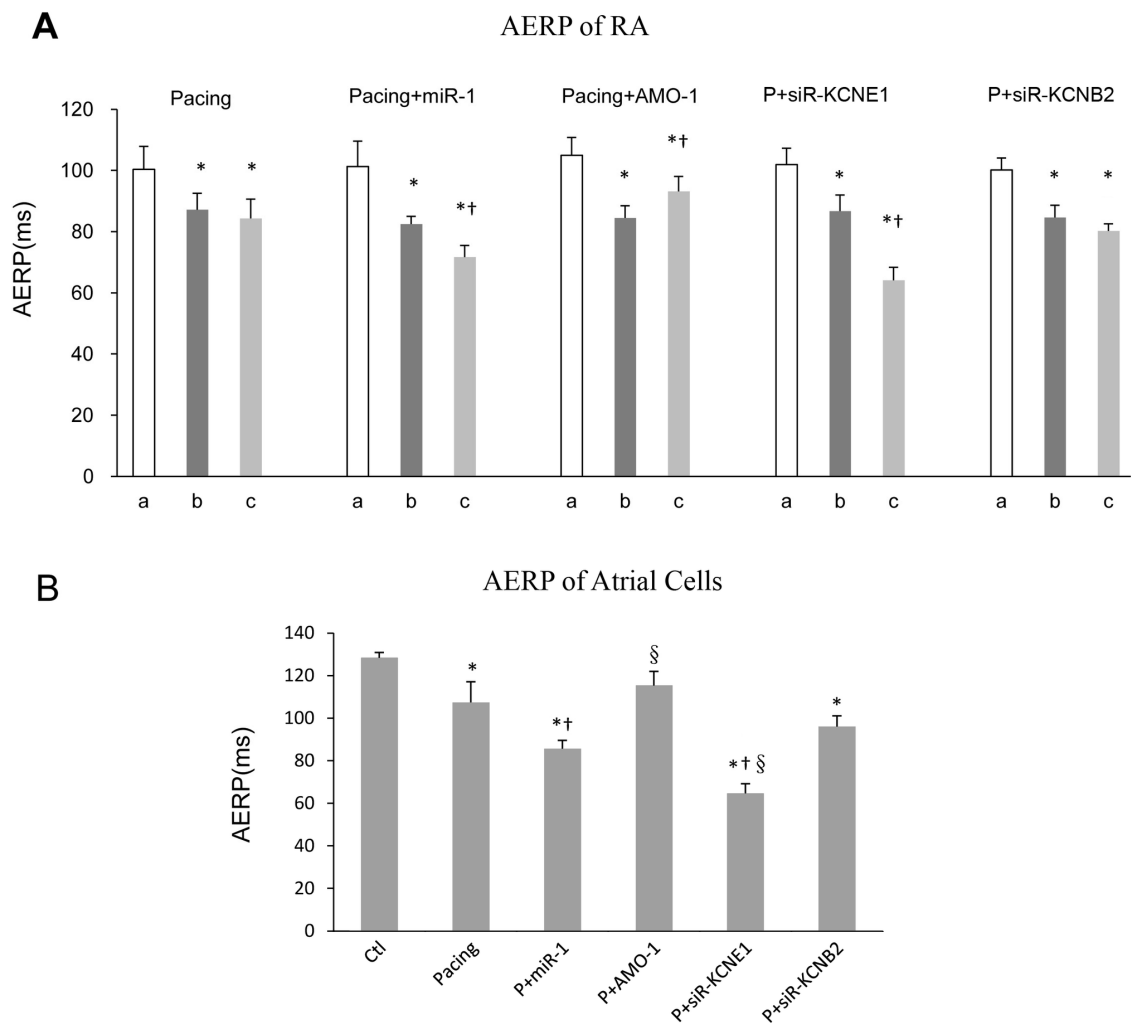
AERP evaluations. AERP of RA obtained from animals as described in the Materials and Methods section (A). The AERP values (*y* axis: ms) were obtained at 3 time points: before pacing (“a”), before infection control/recombinant LVs (“b”), and 1-week after infection (“c”). * *P* < 0.05 vs. “a”; † *P* < 0.05 vs. “b”, one-way ANOVA and Tukey’s post-hoc test; n = 6 independent rabbits for each group. AERP of atrial cells were measured by whole cell patch clamp *in* *vitro* (B).* *P* < 0.05 vs. Ctl, † *P* < 0.05 vs. Pacing, §*P* < 0.05 vs. P+miR-1, one-way ANOVA and Tukey’s post-hoc test; n = 6 independent RNA samples for each group.

**Figure 4:**
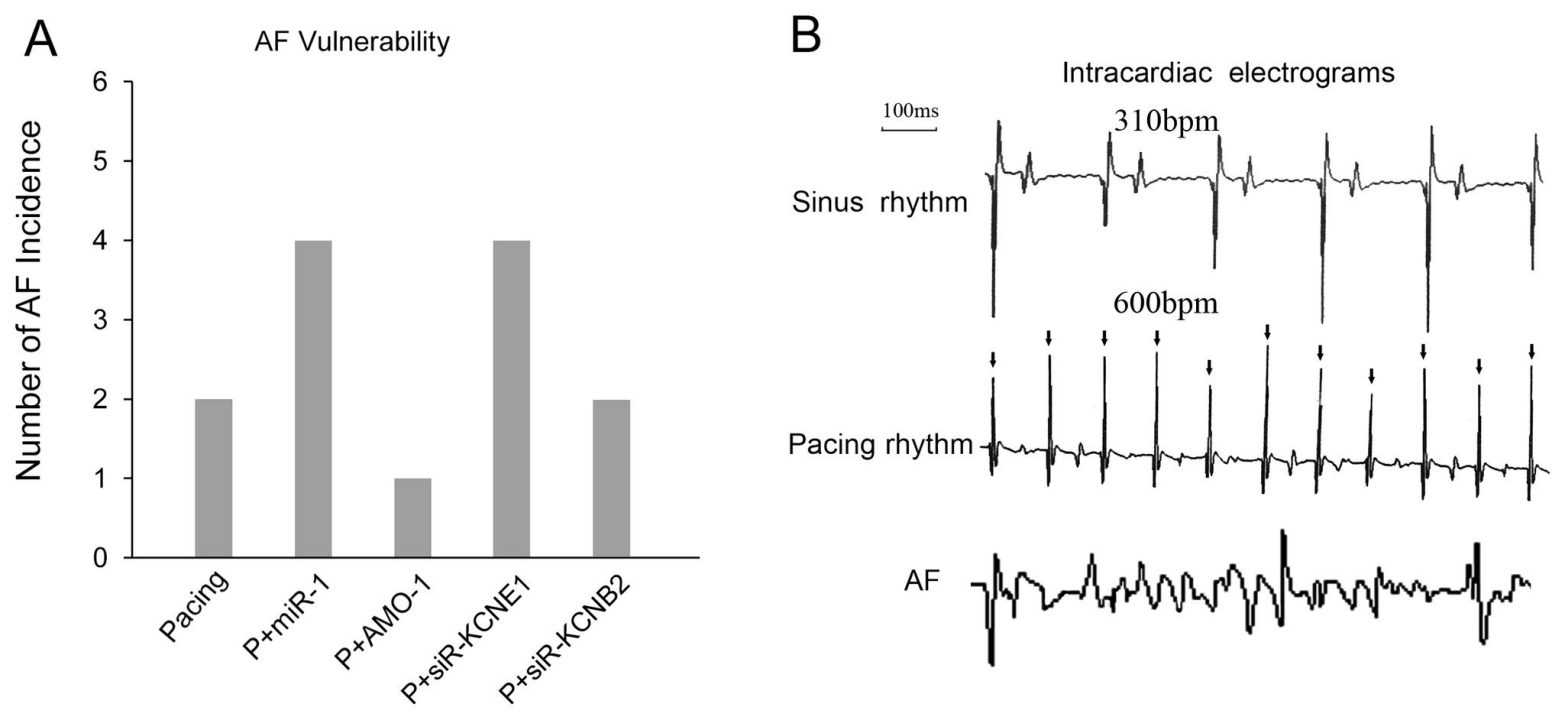
AF inducibility evaluation. The AF inducibility in each group (A). The intracardiac ECGs (B) were obtained from the following sources: (upper ECG) sinus rhythm from the no-pacing rabbits; and (middle ECG) pacing rhythm from the rabbit with pacing; and (lower ECG) AF rhythm from the rabbit with induced AF.

**Figure 5:**
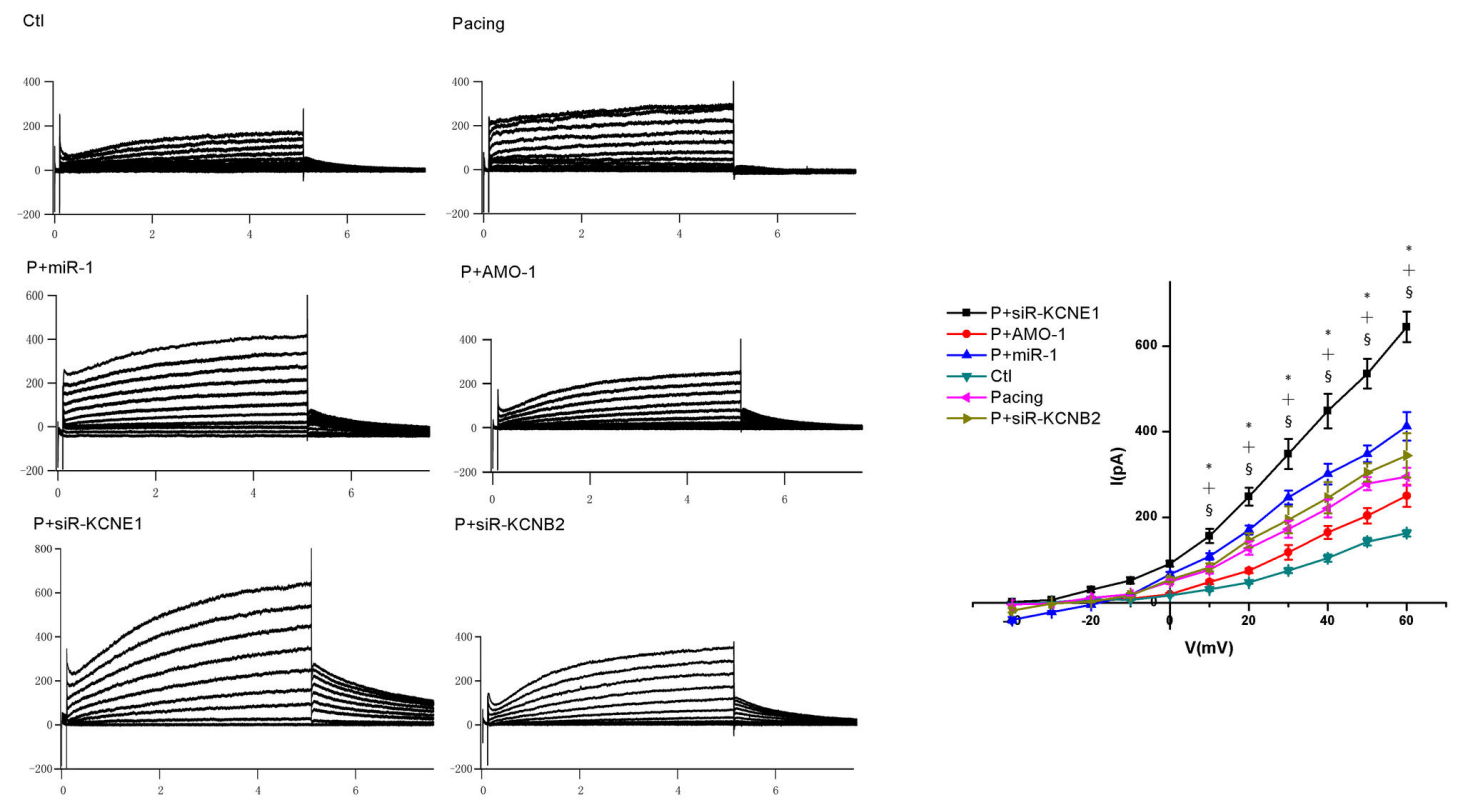
IKs evaluations. The *IKs* in atrial cells was measured using whole cell patch clamp *in* *vitro*. * *P* < 0.05 Pacing vs. Ctl, † *P* < 0.05 P+miR-1 vs. Pacing, § *P* < 0.05 P+siR-KCNE1 vs. Pacing, one-way ANOVA and Tukey’s post-hoc test; n = 6 independent RNA samples for each group.

First, we investigated the effects of A-TP on *miR-1* levels and observed an approximately 1.6-fold increase after pacing (Figure 1A). Second, we selected 22 genes that encoded *K* ^+^ channel proteins and measured their mRNA expression using qRT-PCR (Table S2). We selected *KCNE1* and *KCNB2* for study based on the principle that miRNAs are negative regulators of their target genes (Table S2). The expression levels of the *KCNE1* and *KCNB2* genes (33 and 132 kDa, respectively), were decreased after 1-week of pacing (mRNA: to approximately 17% and 42% less than control levels, respectively) (Figure 2). Third, we studied the effect of A-TP on atrial electrophysiological parameters and AF vulnerability. Animals were processed according to the A-TP model described in the Materials and Methods section, and the AERP of RA was measured using S1-S2 PES at the following 3 time points *in vivo*: before pacing (“a” in Figure 3A), before infection control/recombinant LVs (“b”), and 1-week after infection (“c”). The AERP and the potassium current (*IKs*) in atrial cells were measured using whole cell patch clamp *in vitro*. The AERP of RA was markedly shorter in the pacing group than in the control group after 1-week of A-TP (87±5 vs. 100±8 ms, *P* < 0.05) (Figure 3A). Consequently, AF was induced in 2 rabbits in the pacing group (Figure 4A), whereas no AF induction was observed in the control group (data not shown). These results were confirmed using whole cell patch clamp *in vitro*. The *IKs* was significantly enhanced, and the AERP in atrial cells was obviously shorter in the pacing group compared with the control group (AERP: 107±10 vs. 128±2 ms, *P* < 0.05) (Figures 3B, 5). 

### The effects of *miR-1*/*AMO-1*

The expression level of *miR-1* was approximately 3-fold higher in the P + *miR-1* group after the injection of recombinant LV-*miRNA*-*1* into the RA compared with the pacing group (Figure 1A). The expression levels of *KCNE1* and *KCNB2* were further downregulated (mRNA: to approximately 45% and 37% of the pacing group, respectively) by *miR-1* overexpression via *in vivo* infection. However, *AMO-1* infection reversed the A-TP-induced upregulation of *miRNA-1* to approximately 49% of the pacing group (Figure 1A), and recovered the expression levels of *KCNE1* and *KCNB2* (mRNA: to approximately 1.2-fold and 1.7-fold of the pacing group, respectively) (Figure 2). The relationships between *miRNA-1* and *KCNE1* and *KCNB*2 revealed that *KCNE1* and *KCNB*2 might be the target genes of *miRNA-1*. 

Electrophysiological studies were also performed after *miR-1*/*AMO-1* injections. The *miR-1* overexpression reduced the AERP of RA in the P + *miR-1* group (72±4 vs. 83±3 ms, *P* < 0.05) (Figure 3A). More AF cases (4/6 vs. 2/6) (Figure 4A) were induced after *miR-1* overexpression, and the duration of AF tended to be prolonged (48, 55, 59, 63 vs. 46, 54 s). *AMO-1* reversed the aforementioned effects of *miR-1*: the AERP of RA was significantly prolonged (93±5 vs. 85±4 ms, *P* < 0.05) (Figure 3A), the vulnerability to AF was reduced (1/6 vs. 2/6) (Figure 4A), and the duration of AF tended to be shortened (45 vs. 46, 54 s). To be in line with above results, our cell patch clamp studies indicated an enhanced *IKs* and a shorter AERP in atrial cells in the P + *miR-1* group compared with the pacing group (AERP: 86±4 vs. 107±10 ms *P* < 0.05), and these changes also be reversed by *AMO-1* injection (Figures 3B, 5).

### The effects of *KCNE1*/*KCNB2*

We silenced the *KCNE1* and *KCNB*2 genes individually using siRNA after 1-week of A-TP to verify the role of *KCNE1* and *KCNB*2. *KCNE1* and *KCNB*2 were reduced (mRNA: to approximately 75% and 71% of the pacing group, respectively) (Figure 2), but *miR-1* levels were unaltered compared with the pacing group (Figure 1). Downregulation of *KCNE1* using siR*-KCNE1* shortened the AERP of RA (64±4 vs. 86±3 ms, *P* < 0.05) and increased the inducibility and duration of AF (4/6 vs. 2/6; 47, 53, 62, 64 vs. 46, 54 s) (Figures 3A, 4A). Accordingly, the whole cell patch clamp studies suggested a greater increase in the *IKs* (Figure 5) and a shorter AERP in atrial cells in the P + *siR-KCNE1* group compared with the pacing group (AERP_:_ 65±4 vs. 107±10 ms, *P* < 0.05) (Figure 3B). However, the AERP of RA tend to be shortened in the P + *siR-KCNB2* group, but this difference was not significant (80±2 vs. 85±4 ms, *P* > 0.05) (Figure 3A), and the AF inducibility was also not changed (2/6 vs. 2/6) compared with the pacing group (Figure 4A). These results were also confirmed using the whole cell patch clamp (Figures 3B, 5).

### *KCNE1* and *KCNB2* as target genes of *miR-1*

**Figure 6:**
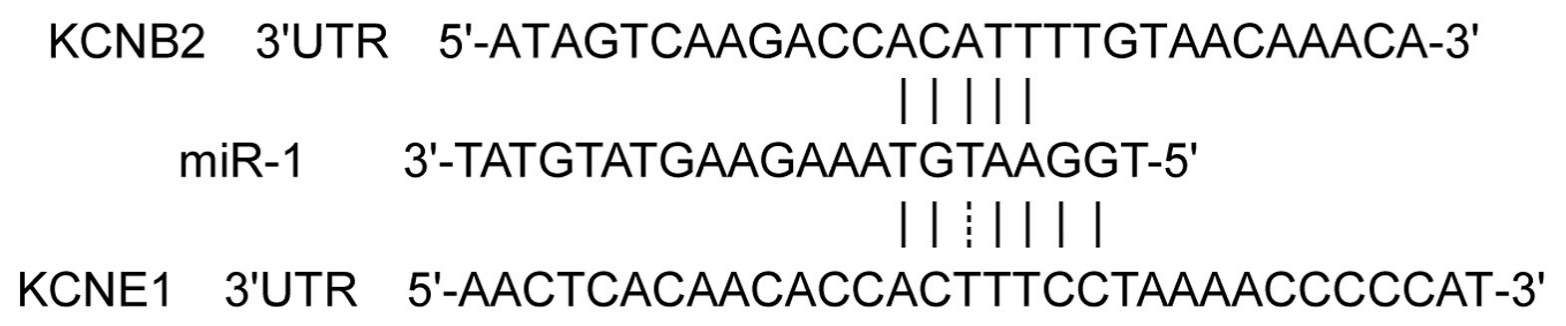
Sequence alignment between miR-1 and putative binding sites in the 3'UTRs of KCNE1 and KCNB2.

**Figure 7:**
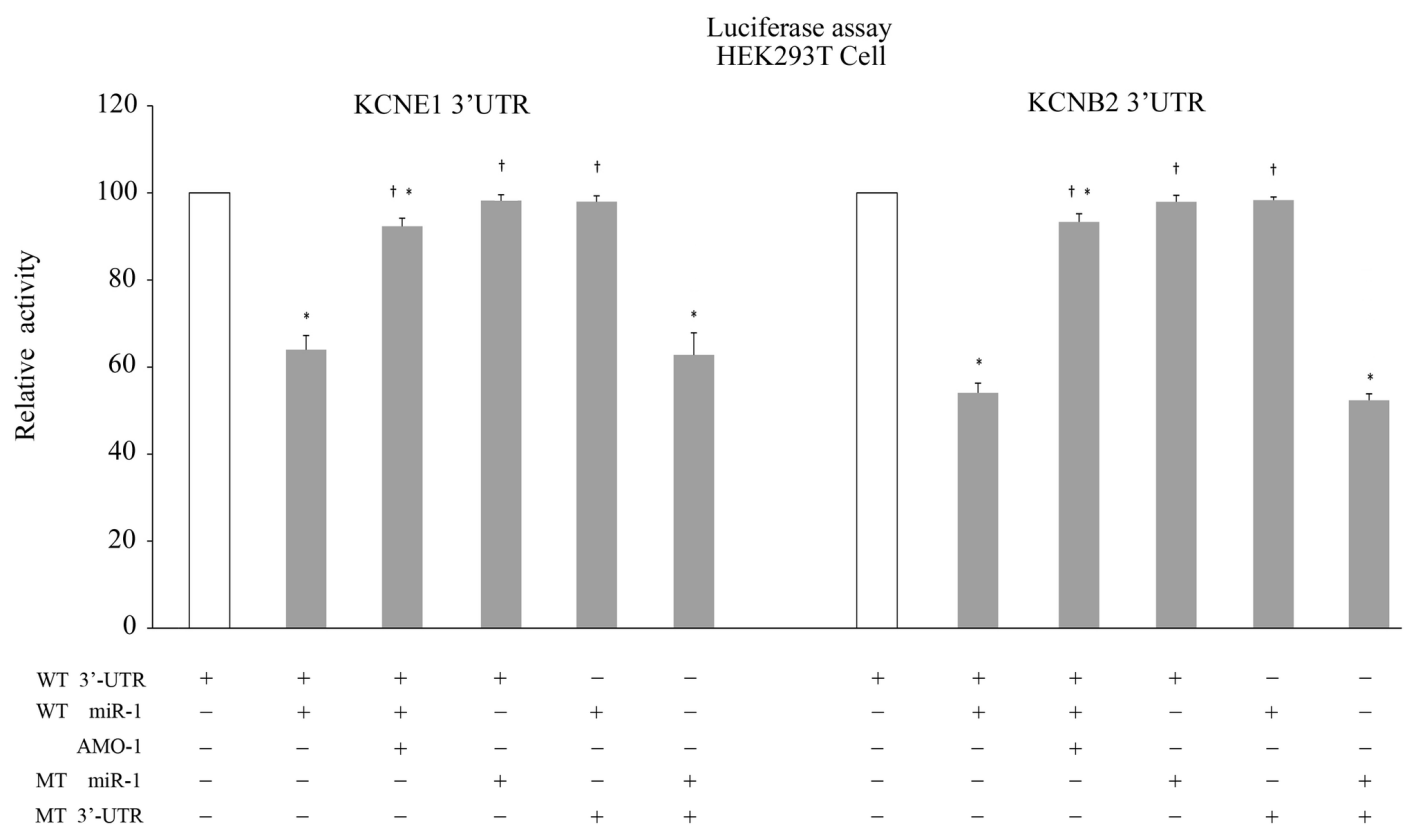
Luciferase assay monitoring of the KCNE1 / KCNB2 - miR-1 interaction. HEK293 cells were co-transfected with *miR-1*- or AMO-1-plasmids and KCNE1 or *KCNB2*- plasmids and the mutation studies by replacing the matching site in *miR-1* and the 3’UTRs of *KCNE1*/*KCNB2* were performed as described in the Materials and Methods section. Luciferase activity, measured in the samples indicated on the “*X*” axis, was normalized to the activity exhibited in the control cells. * *P* < 0.05 vs. Ctl, † *P* < 0.05 vs. miR-1, one-way ANOVA and the Tukey’s post-hoc test.

At least 5 target sites at the 3'UTR of *KCNE1* and *KCNB2* were identified that matched exactly with the 2–8 nucleotides of the 5'-end of *miR-1* (Figure 6). We addressed whether *miR-1* directly regulated *KCNE1* and *KCNB2* using the luciferase reporter assay. The co-infection of HEK293T cells with *miR-1* and the 3’UTRs of *KCNE1*/*KCNB2* plasmids resulted in lower luciferase activity compared with infection with the plasmid alone. However, the inclusion of the *AMO-1*-plasmid in the infection mixture eliminated the silencing effects of *miR-1*. We performed the mutation studies by replacing the matching site in *miR-1* and the 3’UTRs of *KCNE1*/*KCNB2* to ensure the specific effects of our constructs. The mutant *miR-1* did not reduce luciferase activity when co-infected with the 3’UTRs of *KCNE1*/*KCNB2*. Accordingly, *miR-1* also failed to reduce the luciferase activity when co-infected with the mutant 3’UTRs of *KCNE1*/*KCNB2*. On the other hand, the mutant *miR-1* suppressed the translation of luciferase transcripts that contained the complementary mutant 3’-UTR of *KCNE1*/*KCNB2* (Figure 7). Therefore, these data confirmed that *KCNE1* and *KCNB2* were the target genes of *miR-1* in rabbits.

## Discussion

Recent studies have demonstrated that miRNAs are associated with several cardiac defects. *MiR-1* has been reported to contribute to various heart abnormalities, including arrhythmias, myocyte proliferation, and cardiac hypertrophy. This study, explored the effects of *miR-1* on the shortening of AERP after 1-week of A-TP in rabbits. The following major findings were obtained from our study: 1) A right A-TP rabbit model induced by a pacemaker for 1-week increased the expression of *miR-1*; 2) A-TP induced a shortening of AERP (*in vivo* and in atrial cells) and a significantly increased *I*_Ks_; 3) The overexpression of *miR-1* via *in vivo* infection of recombinant LV-*miRNA*-*1* accelerated the shortening of AERP and the increasing of *IKs*, and this effect was reversed by *AMO-1*; 4) *KCNE1* and *KCNB2* as the target genes for *miR-1* were confirmed by the luciferase activity assay.

The role of *miR-1* in AF has been studied previously, but the results are controversial. Girmatsion et al [19]. obtained left atrial (LA) tissue from 62 patients (including 31 with AF) undergoing mitral valve repair or bypass grafting, and found that the *miR-1* level was greatly reduced in AF patients, which may have contributed to the upregulation of the *KCNJ2* and *Kir2.1* subunits. A different result was observed in a later study [15] in which miRNA transcriptomes were obtained from AF patients with rheumatic heart disease and LA samples of dogs with AF that was established using 8 weeks of right A-TP. Microarray analysis and RT-PCR verification revealed no significant changes in *miR-1* levels in either the canine model or AF patients, which is consistent with those of Cooley et al. [20]. We observed increased *miR-1* expression in 1-week right A-TP rabbits. The discrepancy in *miR-1* expression may be due to the following reasons: 1) The results of AF studies induced by A-TP should not be extrapolated to different species; 2) MiRNAs exhibit temporally-specific expression, and the same miRNA may play different roles in different phases and types of AF; 3) The expression of miRNAs may be spatially-specific, therefore, difference could be observed between LA and RA tissue; and 4) The expression of miRNAs in a given tissue may also depend on pathophysiological context, such as metabolic status, disease state, and the pathological conditions of AF.

This study confirmed that the *miR-1* level is upregulated after 1-week A-TP, with corresponding changes in the vulnerability to AF, AERP and *IKs*, thereby implying that *miR-1* may play an important role in atrial ER. Furthermore, the luciferase reporter assay confirmed that *KCNE1* and *KCNB2* were the target genes for *miR-1* in our study*. KCNE1* and *KCNQ1* encode slowly activating delayed rectifier *K* ^+^ current channel, *IK*s, which acts as a powerful repolarization reserve or safety factor to restrict excessive cardiac action potential duration (APD) and QT interval caused by other factors [21-23]. *KCNB2* plays a pivotal role in cell excitability because this gene encodes a member of voltage-gated *K* ^+^ channels that plays an important role in the regulation neurotransmitter release, insulin secretion, neuronal excitability, epithelial electrolyte transport, smooth muscle contraction, and other functions [24-28]. *KCNB2* is expressed in the heart and affects cardiac *K* ^+^ currents [29], but the extensional process remains unclear. 

It was generally believed that the repression of *K* ^*+*^ channel genes, which are responsible for the major outward currents in cardiac repolarization, should lead to a prolonged APD and AERP. However, our research found that the upregulation of *miR-1* reduced in *KCNE1* and *KCNB2* expression, shortened the AERP of RA and enhanced the inducibility of AF. We performed the whole cell patch clamp studies to verify our results *in vivo*. The results show that the *IKs* was indeed enhanced, and the AERP of atrial cells was definitely shortened. We further silenced *KCNE1* using siRNA after 1-week of A-TP while keeping *miR-1* level were unaltered and found that a shorter AERP (*in vivo* and in atrial cells) and significantly increases in the *IKs* were the primary effects of *miR-1* overexpression. There were also many studies supporting our results. Temple et al. [30], demonstrated that: *K* ^+^ currents (total currents and those sensitive to the *IKCNQ1*/*IKs* blocker chromanol 293B) were significantly higher in atrial cells from the mice with deletion of the *KCNE1* gene than in controls, and when CHO cells were driven at very rapid rates (comparable to the normal mouse heart and to human AF), the sigmoidicity displayed by the activating *IKs* results in much less current accumulation compared with *I*_*KCNQ1*_ alone. These authors concluded that *KCNE1* deletion in mice unexpectedly leads to increased outward currents in atrial myocytes, shortens atrial APD, and enhances vulnerability to AF. Heerdt et al. [31] divided young adult Sinclair swines into a ‘control’ group and a ‘postop’ group, with the latter group undergoing lung lobectomy. The AERP was decreased three days after surgery, the vulnerability to AF was increased, and *KCNE1* was down-regulated. We hypothesized that *KCNE1* is an adjusting-subunit of *KCNQ1*, and *KCNE1* played a physiological role via the regulation of *KCNQ1*. The interaction between *KCNE1* and *KCNQ1* is complex. *KCNE1* may negatively regulate the function of *KCNQ1*, but this hypothesis requires further investigation. We also found that *KCNB2* was the target gene for *miR-1*. Most research on *KCNB2* focused on its effects on the nervous system [32,33]. Few studies have explored the effects of *KCNB2* on the cardiovascular system. Our studies indicated that *KCNB2* might exert less important effect than *KCNE1* on the A-TP-induced shortening of AERP. Therefore, we suggest that *miR-1* accelerated the shortening of AERP in rabbits by targeting *KCNE1* and *KCNB2*. 

In conclusion, *miR-1* overexpression accelerated the shortening of AERP induced by A-TP in rabbits, and the knockdown of *miR-1* reversed this shortening via the targeting *K* ^*+*^ channel genes *KCNE1* and *KCNB2*. These results suggest that *miR-1* plays an important role in the ER of AF. Therefore, *miR-1* exhibits significant clinical relevance as a potential therapeutic target for AF. 

### Potential limitations

Our study provides novel insight into the role of *miR-1* in A-TP-induced AERP shortening, but it also has some limitations. First, *KCNE1* and *KCNB2* could not be knocked down at the single-cell level to investigate the changes of *IKs*, and AERP in atrial cells because of the low cell viability after viral infection and the lack of the specific *KCNE1* and *KCNB2* inhibitors in rabbits. Second, the *K* ^+^ current generated by the *KCNB2*-encoded channel was not measured because the knowledge of *KCNB2* is limited presently. Our studies indicated that *KCNB2* might play a possible role in A-TP-induced AERP shortening, but the potential mechanism requires further investigation. 

## Supporting Information

Table S1 **Hemodynamic parameters.**
(DOC)

Table S2 **The expression of mRNA among the 4 groups.**
(DOC)
