# Supplementary material for: MicroRNA-1 Accelerates the Shortening of Atrial Effective Refractory Period by Regulating KCNE1 and KCNB2 Expression: An Atrial Tachypacing Rabbit Model
Source: PLoS One. 2013 Dec 30;8(12):e85639. doi: 10.1371/journal.pone.0085639 (PMC3875574; doi:10.1371/journal.pone.0085639)
Supplement: Table S1 — Hemodynamic parameters. (DOC) [file pone.0085639.s001.doc]

Table S1: Hemodynamic parameters

***Hemodynamic parameters were assessed by heart catheterization 1-week after lentiviral injections to demonstrate the stability of our experiment, and no significant differences between the 6 groups were observed (P < 0.05). HR, heart rate; SBP, systolic blood pressure; DBP, diastolic blood pressure; LVSP, left ventricular systolic pressure; LVEDP, left ventricular end-diastolic pressure.***

| Group | HR | SBP | DBP | LVSP | LVEDP |
| --- | --- | --- | --- | --- | --- |
|  | (bpm) | (mmHg) | (mmHg) | (mmHg) | (mmHg) |
| Ctl | 260±24 | 124.17±7.86 | 89.50±5.19 | 126.17±6.36 | 4.08±0.34 |
| Pacing | 284±33 | 116.67±4.96 | 82.50±5.62 | 118.50±8.24 | 4.22±0.19 |
| P+miR-1 | 281±25 | 119.33±4.71 | 83.83±8.21 | 120.67±3.59 | 4.18±0.16 |
| P+AMO-1 | 279±20 | 117.33±5.62 | 82.83±7.10 | 118.50±8.24 | 4.20±0.24 |
| P+siR-KCNE1 | 287±25 | 114.67±5.62 | 85.17±6.72 | 114.67±3.86 | 4.17±0.12 |
| P+siR-KCNB2 | 281±21 | 118.17±3.67 | 84.17±6.34 | 119.5±6.92 | 4.22±0.20 |
