# Supplementary material for: MicroRNA-1 Accelerates the Shortening of Atrial Effective Refractory Period by Regulating KCNE1 and KCNB2 Expression: An Atrial Tachypacing Rabbit Model
Source: PLoS One. 2013 Dec 30;8(12):e85639. doi: 10.1371/journal.pone.0085639 (PMC3875574; doi:10.1371/journal.pone.0085639)
Supplement: Table S2 — The expression of mRNA among the 4 groups. (DOC) [file pone.0085639.s002.doc]

Table S2: The expression of mRNA among the 4 groups.

We selected *KCNE1* and *KCNB2* for study based on the principle that miRNAs are negative regulators of their target genes. ↑, up-regulation; ↓, down-regulation; -, no change

| **Gene** | **the expression of mRNA** |  |  |
| --- | --- | --- | --- |
|  | Pacing VS Ctl | Pacing VS P+miR-1 | Pacing VS P+AMO-1 |
| **HCN1** | ↑ | ↑ | ↑ |
| **HCN2** | - | - | ↑ |
| **HCN3** | ↑ | ↑ | ↑ |
| **HCN4** | ↑ | ↓ | ↑ |
| **KCNA1** | ↑ | ↑ | ↑ |
| **KCNA3** | ↑ | - | ↑ |
| **KCNA4** | ↑ | ↓ | ↑ |
| **KCNA5** | ↑ | ↓ | ↓ |
| **KCNAB1** | ↑ | ↓ | ↓ |
| **KCN AB2** | ↑ | ↑ | ↑ |
| **KCNB1** | ↓ | ↑ | ↑ |
| **KCNB2** | ↓ | ↓ | ↑ |
| **KCNC1** | ↑ | ↓ | ↑ |
| **KCNC3** | - | - | ↑ |
| **KCND1** | ↓ | ↑ | ↑ |
| **KCND2** | ↑ | ↓ | ↑ |
| **KCND3** | ↑ | ↑ | - |
| **KCNE1** | ↓ | ↓ | ↑ |
| **KCNE2** | - | ↓ | ↑ |
| **KCNH2** | ↑ | ↑ | ↑ |
| **KCNK1** | ↑ | ↑ | - |
| **KCNJ2** | ↑ | ↑ | ↑ |
